# High molecular diversity of full-length genome sequences of zucchini yellow fleck virus from Europe

**DOI:** 10.1007/s00705-022-05558-9

**Published:** 2022-08-09

**Authors:** Kyriaki Sareli, Stephan Winter, Elisavet Κ. Chatzivassiliou, Dennis Knierim, Paolo Margaria

**Affiliations:** 1 Leibniz-Institute DSMZ, German Collection of Microorganisms and Cell Cultures, GmbH, Braunschweig, Germany; 2 Laboratory of Plant Pathology, Department of Crop Science, School of Agricultural Production, Infrastructure and Environment, Agricultural University of Athens, Athens, Greece

## Abstract

Zucchini yellow fleck virus (ZYFV), genus *Potyvirus,* is the causal agent of a disease of cucurbits. The genome sequences of seven ZYFV isolates of different origin were determined, two of which were reconstructed from a squash (*Cucurbita* sp.) collected in 2017 in Greece, while the others, accessions from the DSMZ Plant Virus Collection, were from samples collected in Italy, Greece, and France in the 1980s and 1990s. A high level of molecular diversity, well dispersed along the genome, was observed, but this was within the limits for assignment of the virus isolates to the same species. P1 was the most diverse gene, and isolates from squash contained an insertion in this gene.

**Supplementary Information:**

The online version contains supplementary material available at 10.1007/s00705-022-05558-9.

The genus *Potyvirus* (family *Potyviridae*) includes many economically important viruses of major crops, causing diseases associated with leaf mosaic, fruit discoloration and deformation, yellowing, and necrosis [1]. Epidemics are driven by aphid vectors, which transmit the viruses in a non-persistent manner. Potyvirus particles have a filamentous morphology, are 680-900 nm long, and contain a single-stranded RNA molecule, approximately ~10 kb in length, coding for a polyprotein that is proteolytically processed into at least 10 functional proteins [2]. For taxonomy purposes, the sequence of the complete polyprotein is used for species demarcation, and identity thresholds (below 76% and 82% at nt and aa level, respectively) are used to establish species within the genus [2, 3].

Zucchini yellow fleck virus (ZYFV) was first described in 1981 in zucchini in Italy [4], and it has since been reported in other countries of the Mediterranean basin, including Greece [5], Lebanon and Syria [6], Israel [7], and France [8], and, more recently, in Iran [9]. Given its transmission by *Myzus persicae* and *Aphis gossypii* [4, 8], which are major pests and virus vectors of cucurbits, further spread of the virus and serious impact was expected. However, the occurrence of ZYFV infection has remained localized, and there have never been reports of significant outbreaks of the virus. Despite its description as a member of a distinct potyvirus species [10], molecular characterization of ZYFV has been so far limited to sequences of a short region of the genome encompassing the CP [10, 11], due to a lack of complete genome sequences.

We conducted studies to provide an inventory of common virus diseases of cucurbits in Greece, testing for watermelon mosaic virus (WMV), papaya ringspot virus (PRSV), Moroccan watermelon mosaic virus (MWMV), and zucchini yellow mosaic virus (ZYMV), which are known to be endemic in Greece [12–14] and spread by several aphid species [15, 16]. When using high-throughput sequencing (HTS) to analyze a squash (*Cucurbita* sp.) sample with virus symptoms that was collected in 2017 in central Greece and had tested positive by ELISA for a potyvirus [17], we discovered two distinct isolates of ZYFV. A comparison with full-length genome sequences of ZYFV isolates from the DSMZ Plant Virus Collection with diverse geographic origin and collection dates (Table 1) revealed a high level of molecular diversity. Notably, the Greek ZYFV isolates from squash contain a distinctive insertion in the P1 coding region, whose possible biological function remains to be determined.

**Table 1:** DSMZ Plant Virus Collection accessions used in this study for reconstruction and characterization of ZYFV genome sequences

DSMZ accession	Country of origin	Year of collection	Host	GenBank accession no.
PV-0379	Italy, Apulia	1980	*Cucurbita pepo*	ON456303
PV-1256	Italy, Sicily	1990	*Cucurbita pepo*	ON456306
PV-1257	Italy, Sardinia	1995	*Cucurbita pepo*	ON456307
PV-1258	Greece	1986	*Cucurbita pepo*	ON456308
PV-1263	France	1987	*Ecballium elaterium*	ON456309
PV-1248	Greece, Farsala	2017	*Cucurbita* sp.	ON456304, ON456305

During field surveys conducted in 2017 in Farsala (Larissa Prefecture, Central Greece), leaf samples from a symptomatic squash showing fleck and yellow pinpoint spots on old and young leaves (Supplementary Fig. S1) were collected, and they tested positive by ELISA using a potyvirus group-specific antibody (DSMZ RT-0573/1) [17]. Further screening with antisera against WMV (DSMZ RT-0203), PRSV (DSMZ RT-0805), ZYMV (DSMZ RT-0234), and MWMV (DSMZ RT-0747) gave negative results. We therefore employed HTS followed by virus discovery analysis using the workflow established at the DSMZ Plant Virus Department [18]. We sequenced the original squash sample for a primary virome investigation and a zucchini plant that was infected mechanically with homogenates of the original sample. Total RNA was extracted from symptomatic leaves, using an RNeasy Plant Mini Kit (QIAGEN) or a Spectrum Plant Total RNA Kit (Sigma-Aldrich). Ribosomal RNA was depleted, followed by random cDNA synthesis and second-strand synthesis using random octamer primers. Nextera XT libraries (Illumina) were prepared according to the manufacturer's instructions and sequenced on a MiSeq or NextSeq instrument as paired-end reads. The reads were assembled *de novo* using Geneious Prime v. 2022.0.1 (Biomatters) and were screened by BLAST against a custom reference virus database to reveal significant hits against potyvirus sequences assigned to ZYFV. To study the genome diversity of ZYFV, the sequences of five virus accessions from the DSMZ Plant Virus Collection were also determined following the same workflow. The analysis included ZYFV isolates from the 1980s and 1990s collected from zucchini in Italy (DSMZ PV-0379, PV-1256, PV-1257) and Greece (DSMZ PV-1258), and from *Ecballium elaterium* in France (DSMZ PV-1263) (Table 1).

Bioinformatic analysis allowed the reconstruction of seven ZYFV genome sequences (Supplementary Table S1), two of which (PV-1248 isolate #1 and #2) were assembled from the zucchini that had been infected mechanically with homogenates from the squash sample collected in 2017. The assembled genome sequences ranged in length from 10,161 to 10,325 nt and contained an open reading frame (ORF) encoding a polyprotein of 3320-3338 aa in length, and they exhibited a genome organization typical of potyviruses (Fig. 1a). The 5´- and 3´-terminal ends of the genome of isolates PV-1256 and PV-1257 were verified by RACE-PCR assays and direct sequencing of the amplification products. Sequence alignment revealed that two isolates, PV-0379 from zucchini from Apulia and PV-1258 from Greece (from 1986), were ~99% identical, indicating introduction and/or exchange of the virus between these two geographically close areas. Given the similarity of the two sequences, we refer to the two isolates as one entity. A high degree of sequence diversity among the isolates was found, evenly distributed over the entire length of the genome. This confirmed the high molecular divergence reported previously for the 3´-terminal region of the genome [10]. Alignment of the polyprotein sequences revealed 75.4-82.8% and 83.6-92.9% identity at the nt and aa level, respectively (Supplementary Table S2), which, according to current guidelines, supports the assignment of these viruses to the same species. The isolate PV-1263 from squirting cucumber (*E. elaterium*) from France had the most divergent sequence (Supplementary Table S2), which may correlate with adaptation to this host, a wild perennial gourd plant. A phylogenetic analysis of the ZYFV genome sequences with those of other potyviruses showed that the ZYFVs form a distinct clade that is well separated from the clade including papaya ringspot virus (PRSV) and zucchini tigré mosaic virus (ZTMV) (Fig. 2). This striking diversity among the ZYFV sequences exceeded the diversity of PRSV isolates, forming a less-dispersed cluster. Pairwise comparison with the coding regions of PRSV revealed an extreme diversification of P1 (~42 and ~28% nt and aa identity, respectively), with highest similarity in the NIb (~69 and ~76%, respectively) followed by HC-Pro (~68 and ~75%, respectively) (Supplementary Table S1). Compared to ZTMV, the P1 gene of ZYFV showed ~43 and ~31% nt and aa identity, respectively, and highest identity was observed with NIb and HC-Pro (Supplementary Table S2).

**Fig. 1:**
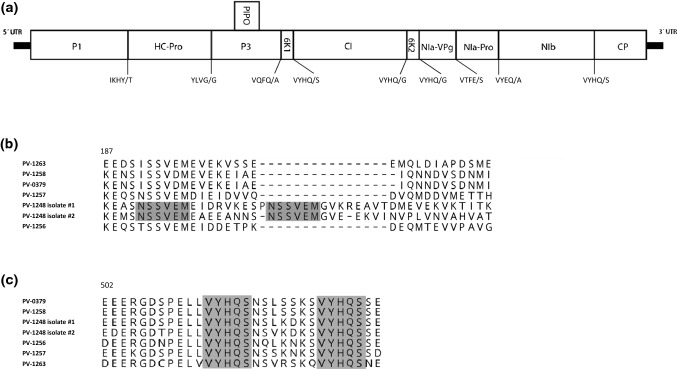
**(**a) Schematic representation of the genomic organization of zucchini yellow fleck virus. The sequence map of PV-1248, isolate #2, assembled from a squash collected in Farsala (Central Greece) and verified by Sanger sequencing, is presented. The putative cleavage sites in the polyprotein are shown. (b) Amino acid sequence alignment of the P1 protein region showing an insertion unique to the Greek ZYFV isolates from squash. The gray box indicates the amino acid sequence motif duplicated in the insertion. (c) Amino acid sequence alignment showing the duplicated putative NIb/CP cleavage site (VYHQ/S), separated by a variable sequence of 7 aa

**Fig. 2:**
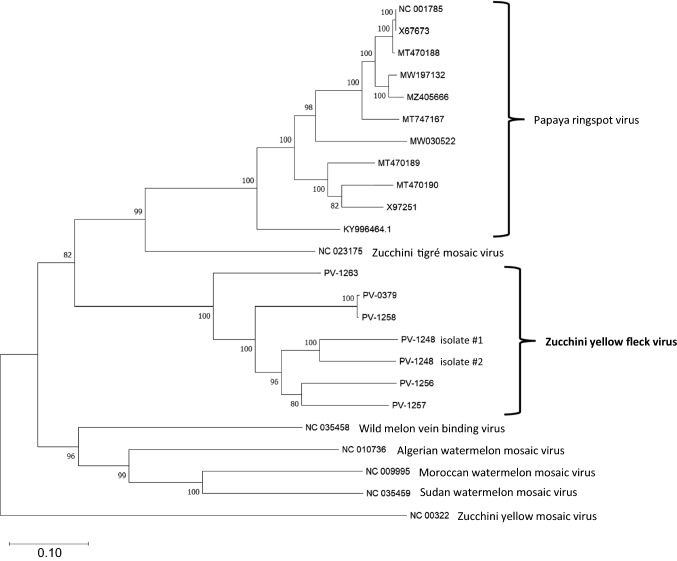
Phylogenetic tree constructed using the seven zucchini yellow fleck virus genome sequences assembled in this study and other potyvirus sequences obtained from the GenBank database. The evolutionary history was inferred using the maximum-likelihood method and the general time-reversible model implemented in MEGA [42]. The scale bar represents a value of 0.01 substitutions per site

The first mature protein, the serine protease P1, is predicted to cleave itself from HC-Pro at residues Tyr/Ser or Tyr/Thr (Supplementary Table S3). Its diversity in sequence and size among potyviruses is well known [19], and P1 was indeed found to be the most variable gene among the ZYFV isolates. The predicted P1 protein contained a highly variable region at position 200-220, with the highest diversity found in the sequences from squash, which included an insertion with the duplication of an “NSSVEM” motif located closely upstream (Fig. 1b). Verification of the ZYFV genome sequence by Sanger sequencing of RT-PCR products (Supplementary Table S4) confirmed this unique feature in the two isolates from squash. Future investigations are needed to clarify whether the insertion is a characteristic motif of current ZYFV populations in Greece and to investigate its possible effect on biological functions. A functional role of the P1 and its diversity in determining the host range has been demonstrated for the potyviruses plum pox virus and tobacco vein mottling virus [20, 21]. Similarly, the P1a protein of cucumber vein yellowing virus, a member of the genus *Ipomovirus* in the *Potyviridae*, is a determinant of host adaptation and host specificity [22, 23]. Coevolution of ZYFV with distinct hosts may explain the observed divergence of the genome sequences from squash isolates from those from zucchini and squirting cucumber (Table 1).

The HC-Pro protease is predicted to consist of 457 aa in all isolates, with the cleavage site YLVG/G in the polyprotein of all seven isolates (Supplementary Table S3). At the N-terminus of HC-Pro, the zinc finger motif HXCX_8_CX_13_CX_4_CX_2_C, starting at position 24, includes the RITC motif, which is involved in aphid transmission of other viruses, including tobacco vein mottling virus [24], MWMV [25], and pepper vein banding virus [26]. The PTK motif, which is also involved in aphid transmission [27], is located at position 309-311. The motifs IGN and CCC, which have been implicated in replication and movement of potyviruses [28–30], are located at aa positions 249-251 and 291-293, respectively.

The remaining viral proteins are produced by cleavage by the cysteine protease NIa-Pro with the typical sequence motifs V-X-[F;H]-[Q;E]/[A;G;S] flanking the cleavage sites (Supplementary Table S3). The proteins 6K1, CI, 6K2, VPg, and NIa-Pro are predicted to be of the same size in all ZYFV isolates. As observed in a previous analysis on aa preferences of NIa-Pro protease in the polyprotein of potyviruses [31], a glutamic acid residue is conserved at position -1 of the VPg/NIa-Pro junction. A duplicated cleavage motif at the NIb/CP junction [10], with the sequence VYHQ/S, is present in all isolates, with an inter-motif variable region of 7 aa (Fig. 1c). A putative second NIb/CP cleavage site has also been reported for ZTMV [32] and PRSV, although in the latter case, the motif is not completely conserved [10]. The biological significance of this feature of the polyprotein remains to be determined.

Pairwise comparisons of the CP sequence showed that PV-1263 from France was the most divergent virus, with ~87-90% aa sequence identity to the other isolates. As has been observed in the CPs of other potyviruses [33–35], most of the divergence was observed in the N-terminal region of the protein, at positions 13-15 and 24-32, while aa residues at positions 121 to 277 were identical among all sequences; a deletion of 3 aa at position 29-31 was unique to PV-1263. A phylogenetic analysis with other CP sequences available in the NCBI/GenBank database showed that the Greek isolates from squash grouped in a cluster with the isolate from Apulia/Greece, while the CP of the DSMZ accessions from Sicily and Sardinia and two sequences from melon collected in 2006 in Sicily [11] formed a separate sequence cluster (Supplementary Fig. S2). All ZYFV genomes encoded a “DAA(A)” motif in the N-terminal region of the CP, instead of the more common “DAG(X)” implicated in aphid transmission [36]. This motif was also reported in the potyviruses onion yellow dwarf virus and peanut mosaic virus [37].

The PIPO ORF, embedded within the P3 region of the polyprotein and translated in the +2 reading-frame [38], was annotated and found to contain the highly conserved G_1-2_A_6-7_ motif in the form GAAAAAA in all sequences.

To assess the role of natural selection at the molecular level, the rate of non-synonymous substitutions per non-synonymous site (dN) and the rate of synonymous substitutions per synonymous site (dS) were calculated for each virus gene using DnaSP v. 6.12.03 [39]. The results revealed that the ZYFV genome is under negative selection, with strong selection in HC-Pro and weak selection in P1 and PIPO (Supplementary Table S5). This is in accordance with common observations for potyviruses [40].

Since recombination is shaping potyvirus populations [40], we performed a recombination analysis with RDP5 [41] on a MUSCLE alignment of the ZYFV genome sequences and included a set of PRSV and ZTMV sequences (Supplementary Table S6). One recombination event in P1 of PV-1256 from Sicily was traced to PV-1248 isolate #1 as the major parent, but the minor parent is unknown (Supplementary Table S6). Two recombination events involved ZYFV and potyviruses of other species, but the recombination signals were only weakly supported. We can thus assume that the ZYFV isolates in this study had not undergone genetic recombination.

In summary, the complete genome sequences of ZYFV isolates from different European countries were determined. Sequence comparisons revealed that the ZYFV isolates from squash from Greece contained a unique motif in the P1 region, the biological role of which remains to be determined.

## Supplementary Information

Below is the link to the electronic supplementary material.

Supplementary Fig. S1 Symptoms on old (a) and young (b) leaves of the Cucurbita sp. plant sampled during a survey conducted in open fields in 2017 in Farsala (Larissa Prefecture, Central Greece)

Supplementary Fig. S2 Phylogenetic tree constructed based on the deduced coat protein aa sequences of zucchini yellow fleck virus isolates from this study (indicated by arrows) and those obtained from GenBank. Sequences were aligned using the MUSCLE algorithm included in MEGA [42]. The evolutionary history was inferred by the maximum-likelihood method and the Jones-Taylor-Thornton matrix-based model, with 500 bootstrap replicates. The scale bar represents a value of 0.01 substitutions per site

Supplementary file3 (TXT 72 KB)

Supplementary file4 (XLSX 11 KB)

Supplementary file5 (XLSX 12 KB)

Supplementary file6 (XLSX 11 KB)

Supplementary file7 (XLSX 12 KB)

Supplementary file8 (XLSX 21 KB)

## Data Availability

The datasets generated during and/or analysed during the current study are available from the corresponding author on reasonable request.
