# Supplementary figures and images for: High molecular diversity of full-length genome sequences of zucchini yellow fleck virus from Europe
Source: Arch Virol. 2022 Aug 9;167(11):2305–10. doi: 10.1007/s00705-022-05558-9 (PMC9556397; doi:10.1007/s00705-022-05558-9)

## Slide 1
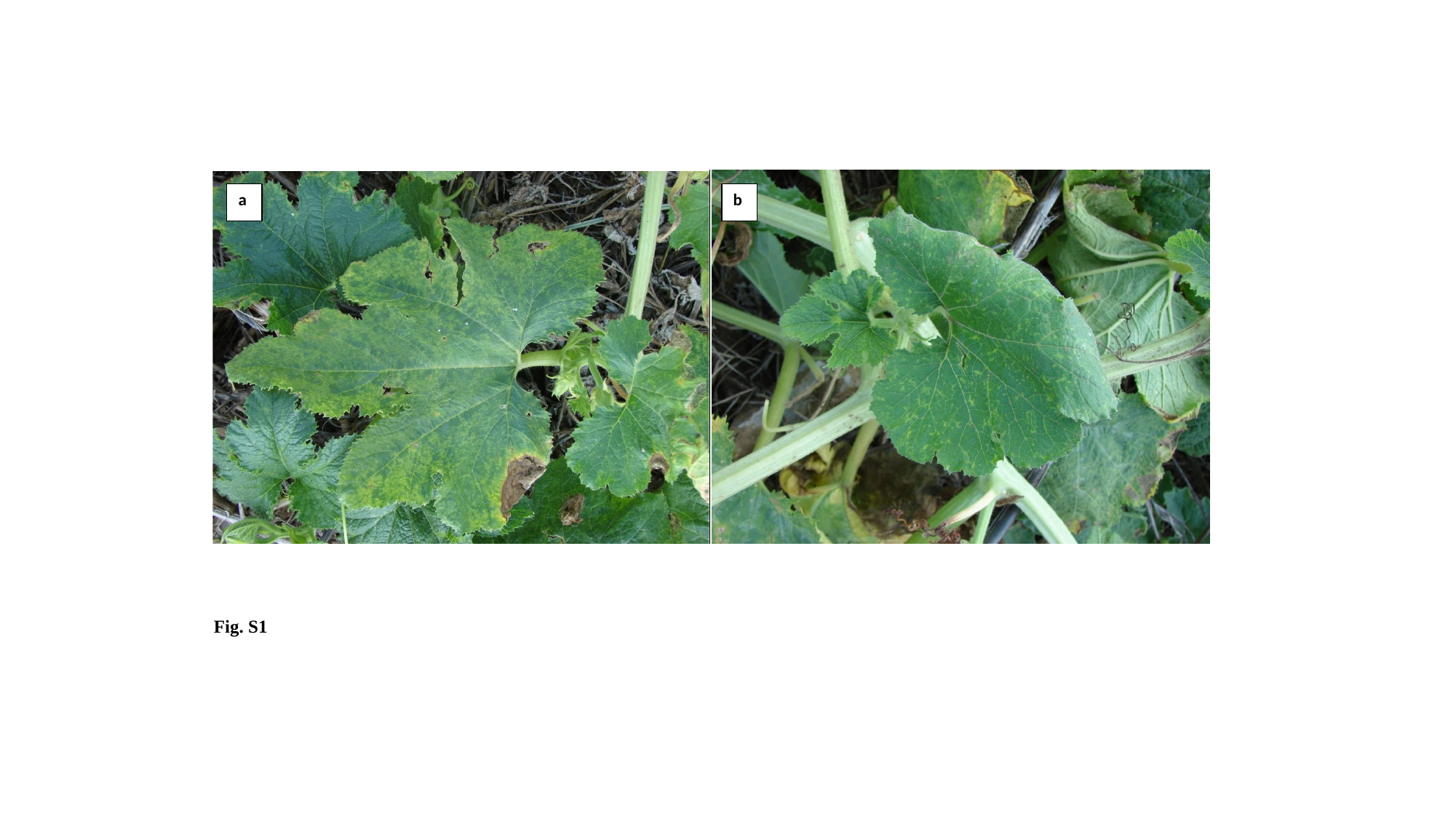

Fig. S1

Supplement: Supplementary file 1 — Supplementary Fig. S1 Symptoms on old (a) and young (b) leaves of the Cucurbita sp. plant sampled during a survey conducted in open fields in 2017 in Farsala (Larissa Prefecture, Central Greece) [file 705_2022_5558_MOESM1_ESM.pptx]
