# Supplementary material for: High molecular diversity of full-length genome sequences of zucchini yellow fleck virus from Europe
Source: Arch Virol. 2022 Aug 9;167(11):2305–10. doi: 10.1007/s00705-022-05558-9 (PMC9556397; doi:10.1007/s00705-022-05558-9)
Supplement: Supplementary file 2 — Supplementary Fig. S2 Phylogenetic tree constructed based on the deduced coat protein aa sequences of zucchini yellow fleck virus isolates from this study (indicated by arrows) and those obtained from GenBank. Sequences were aligned using the MUSCLE algorithm included in MEGA [42]. The evolutionary history was inferred by the maximum-likelihood method and the Jones-Taylor-Thornton matrix-based model, with 500 bootstrap replicates. The scale bar represents a value of 0.01 substitutions per site [file 705_2022_5558_MOESM2_ESM.pptx]

## Slide 1
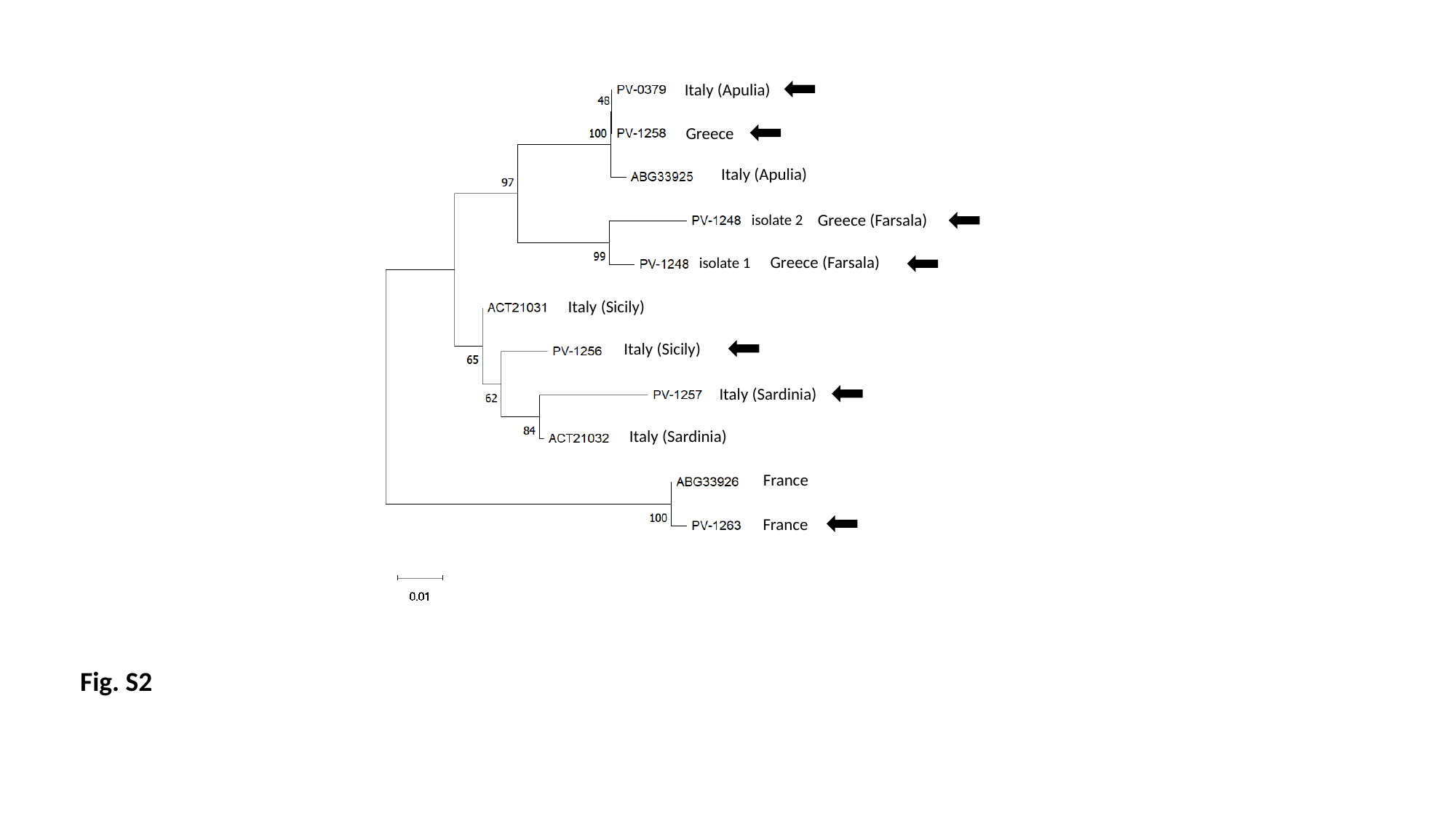

Italy (Apulia)
Greece
Italy (Apulia)
Greece (Farsala)
Greece (Farsala)
Italy (Sicily)
Italy (Sicily)
Italy (Sardinia)
Italy (Sardinia)
France
France
isolate 2
isolate 1
Fig. S2
